# A comparative study using dual-energy X-ray absorptiometry, air displacement plethysmography, and skinfolds to assess fat mass in preterms at term equivalent age

**DOI:** 10.1007/s00431-020-03812-3

**Published:** 2020-10-01

**Authors:** Dana F. J. Yumani, Dide de Jongh, Harrie N. Lafeber, Mirjam M. van Weissenbruch

**Affiliations:** 1 Department of Pediatrics, Amsterdam UMC, Location VU University Medical Center, De Boelelaan 1117, 1081 HV Amsterdam, The Netherlands; 2 Faculty of Science, Vrije Universiteit Amsterdam, Amsterdam, The Netherlands

**Keywords:** Fat mass, Dual-energy X-ray absorptiometry, Air displacement plethysmography, Skinfold thickness, Premature infants

## Abstract

The aim of this study was to compare whole body composition, generated by air displacement plethysmography (ADP) and dual-energy X-ray absorptiometry (DXA), and to evaluate the potential predictive value of the sum of skinfolds (∑SFT) for whole body composition, in preterm infants at term equivalent age. A convenience sample of sixty-five preterm infants with a mean (SD) gestational age of 29 (1.6) weeks was studied at term equivalent age. Fat mass measured by DXA and ADP were compared and the ability of the ∑SFT to predict whole body fat mass was investigated. There was poor agreement between fat mass percentage measured with ADP compared with DXA (limits of agreement: − 4.8% and 13.7%). A previously modeled predictive equation with the ∑SFT as a predictor for absolute fat mass could not be validated. Corrected for confounders, the ∑SFT explained 42% (ADP, *p* = 0.001) and 75% (DXA, *p* = 0.001) of the variance in fat mass percentage.

*Conclusions*: The ∑SFT was not able to accurately predict fat mass and ADP and DXA did not show comparable results. It remains to be elucidated whether or not DXA provides more accurate assessment of whole body fat mass than ADP in preterm infants.

*Trial registration*: NTR5311

**Table:** 

**What is Known:** • *Diverse methods are used to assess fat mass in preterm infants.*	
**What is New:** • *This study showed that there is poor agreement between dual-energy X-ray absorptiometry, air displacement plethysmography, and skinfold thickness measurements.* • *Our results affirm the need for consensus guidelines on how to measure fat mass in preterm infants, to improve the assimilation of data from different studies and the implementation of the findings from those studies.*

**What is Known:**

• *Diverse methods are used to assess fat mass in preterm infants.*

**What is New:**

• *This study showed that there is poor agreement between dual-energy X-ray absorptiometry, air displacement plethysmography, and skinfold thickness measurements.*

• *Our results affirm the need for consensus guidelines on how to measure fat mass in preterm infants, to improve the assimilation of data from different studies and the implementation of the findings from those studies.*

## Introduction

Preterm infants are prone to develop risk factors for the metabolic syndrome in later life [1]. Adolescents and adults born preterm have been shown to have a higher fat mass, a higher blood pressure, and an increased risk of dysglycemia compared with adolescents and adults born at term [2, 3]. While some report no differences in fat distribution at younger ages [4], others did find difference in infancy when comparing the body composition of infants born preterm with that of those born at term [5]. For instance, at term equivalent age, premature infants have been reported to have an increased fat mass compared with term infants [5]. Term equivalent age is an important benchmark for the development of the preterm infant: a point to evaluate whether any disparities in extra-uterine development and normal fetal development bear short- or long-term consequences. Since in adulthood the fat mass percentage and the fat mass index have been related to the occurrence of metabolic syndrome components [6, 7], monitoring body composition in infancy and childhood could help to signal early signs of increased disease risk. Therefore, to ensure the timely implementation of preventive measures, it is pertinent to have a validated method to assess body composition, in particular fat mass.

The most frequently used methods to estimate fat mass are air displacement plethysmography (ADP) and dual-energy X-ray absorptiometry (DXA). There is no consensus on which reference method should preferentially be used and at the same time studies in term infants show poor agreement between fat mass measured with ADP compared with DXA [8–10]. To our knowledge, there is no published data on the comparison of ADP and DXA in preterm infants. Therefore, the purpose of the present study was to compare DXA-generated and ADP-generated whole body composition in preterm infants at term equivalent age. Even so, ADP and DXA are both expensive and immobile instruments. Therefore, it would be valuable to have a reliable and low-cost point-of-care instrument. Skinfold measurements have been suggested as a low budget tool for measuring fat mass in infants, in particular in low-income countries [11, 12]. Nevertheless, there are questions about the reliability and reproducibility of skinfold measurements. Moreover, despite several studies assessing predictive equations including skinfolds or the sum of skinfolds (∑SFT) to estimate fat mass, to our knowledge, only a few included preterm infants [12–15]. In addition, the limited publications on the predictive value of SFT for fat mass percentage in preterm infants included mainly late preterm infants [16]. All in all, at this time, there are no validated predictive equations including SFT for extremely and very preterm infants. Therefore, this study assessed the potential predictive value of the ∑SFT for fat mass and fat mass percentage in preterm infants. In conclusion, the aim of this study was to assess the agreement between fat and fat-free mass measured with ADP and DXA and estimated by the sum of skinfolds, in preterm born infants at term equivalent age.

## Methods

### Study cohort

The study cohort consisted of a convenience sample of 65 preterm infants born between 2015 and 2018, with a gestational age of 24 to 32 weeks, admitted to the neonatal intensive care unit of the Amsterdam University Medical Centers, location Vrije Universiteit University Medical Center. The preterm infants were part of the NUTRIE study, a longitudinal observational study on nutrition in relation to the endocrine regulation of preterm growth and body composition. The NUTRIE study was powered to detect a medium size effect (*r* = 0.35) of insulin-like growth factor 1 on fat mass percentage. No power calculations were done for the primary outcomes presented in this paper. To demonstrate that the maximum allowed difference in fat mass measured by two different methods is < 200 g, 10 pairs would be needed based on a mean difference in the population of 100 g (± 25) [8].

Informed consent was obtained in the first week of life and participants were followed up from birth to 2 years corrected age. Infants with substantial congenital anomalies based on a chromosomal disorder or syndrome were excluded.

The study was approved by the medical research ethics committee of the Vrije Universiteit University Medical Center and was conducted according to the good clinical practice guidelines and in line with the Declaration of Helsinki. The study was registered at the Dutch Trial Register where an audit trail of changes to the design was kept (www.trialregister.nl; NTR5311).

### Assessment of growth and body composition

Growth and body composition were assessed on the same day in the same order in all participants. Follow-up at term equivalent age was planned between 38 and 46 weeks postmenstrual age (mean 43.8 ± 1.9 weeks). SFT were measured first, followed by ADP and finally DXA. Infants were fed before the DXA in case the child was too agitated.

Growth was assessed from birth until 36 weeks postmenstrual age and at term equivalent age. Measurements of weight, length, and head circumference were taken by two investigators. Infants were weighed nude on an electronic scale to the nearest 5 g and length was measured with a length board to the nearest 0.5 cm. Occipital-frontal head circumference was measured to the nearest 0.1 cm with a non-stretchable measuring tape. Standard deviation scores (SDS) of weight, length, and head circumferences were calculated according to Fenton [17]. Small for gestational age (SGA) was defined as a birth weight below the tenth percentile (− 1.3 SD) and postnatal growth restriction was assumed if, at 36 weeks postmenstrual age, there was a decrease in weight *z*-score of more than 1 SD compared with the birth weight *z*-score [18].

Skinfolds were measured (to the nearest millimeter) at biceps, triceps, subscapular, and supra-iliac positions with a Harpenden® skinfold caliper by two investigators. One measurement was taken bilaterally for every position. The bilateral measurements were averaged to come to one skinfold thickness for every position. According to previous studies, the intraobserver coefficient of variation is below 3%; however, the inter-observer coefficient is up to 10% [15].

The anthropometric formula which was used to estimate fat mass at term equivalent age was that of Schmelzle and Fusch [12]. This formula was originally modeled to predict fat mass, measured with DXA, in infants 34 weeks gestational age and older, using ∑SFT (mm) and length (cm): fat mass (g) = 68.2 × ∑SFT ^ (0.0162 × length) − 172.8. Skinfolds were measured at the same site as our study. This formula was selected because it was the only predictive equation based on a population that included preterm infants and gave a high explanation of the variance in fat mass [12, 14].

The Pea Pod® (PEA POD Infant Body Composition System, Cosmed Ltd, Concord, CA, USA) was used to assess whole body fat mass and fat-free mass through ADP. The measurements were performed by two investigators. Infants were measured naked and hair was flattened using hair oil. Infants were allowed to move during the measurement. In case of excessive crying, the measurement was stopped. Measurements were done briefly before feeding time, i.e., approximately 3 h after the last feeding. In line with the manufacturer’s guideline, daily quality control checks were done which included chamber calibration. Every 2 weeks, the scale was calibrated. A detailed description of the Pea Pod® measurement is described elsewhere [19]. As previously reported, the coefficient of variation for repeated volume measurements lies between 0.02 and 0.09% [19]. Fat mass and fat-free mass were calculated using gender-specific equations developed by Fomon and colleagues [20].

The Hologic QDR 4500 A, using Infant Whole Body Software version 13.5.3:3 (Hologic Inc., Bedford, MA, USA), was used to assess whole body fat mass and fat-free mass through dual-energy X-ray absorptiometry. During the procedure, the infants were required not to move. The infants were swaddled in a blanket of the same size and type supplied by the investigators, without any clothing or diapers. Infants were swaddled in supine position with the soles of the feet together and knees bent (frog-leg-position) and the arms stretched beside the body. Infants were positioned in the center of the scanning bed with their head near the head end of the bed. The measurement was done after feeding. Typically infants remained awake, but lights were dimmed and a video was played from a mobile device outside of the scanning field. The preparation and positioning of the infants were performed by two experienced investigators. Calibration was done daily using an anthropomorphic spine phantom and a geometric block phantom. In addition, a radiographic uniformity test was done once a week and software was regularly updated. All images were analyzed by one radiologist. Images with excessive movement artifacts were excluded at the judgment of the radiologist.

### Potential confounders

The following factors are known to relate to body composition and were assessed as potential confounders: gestational age, gender, ethnicity, type of nutrition: human milk (60% or more of total diet) vs formula (60% or more of total diet), waist circumference and absolute weight, length and head circumference at birth and term age, and their corresponding *z*-scores [15].

### The statistical analysis

Characteristics of the study group were first summarized using descriptive statistics, stratified by sex. Mean and standard deviations (SD) were calculated for all continuous variables and presented as mean ± SD. Percentages were reported for dichotomous variables. The median and the interquartile range were reported if the variable was not normally distributed.

The level of agreement and potential bias between fat mass percentage obtained via ADP and DXA was examined using the Bland-Altman analysis [12]. Agreement between the formula of Schmelzle et al. [12] and fat mass was also examined using Bland-Altman plots. Based on the normal variation in fat mass, the maximum allowed difference was set at 200 g for absolute fat mass and at 2% for fat mass percentage [21–23].

Prediction models were developed for predicting the variable ∑SFT and absolute fat mass and fat mass percentage measured with ADP at term equivalent age. Potential confounders which showed significant correlations with fat mass (percentage) in univariate analysis were added together in a multivariate model. The final model was determined through a backward stepwise regression analysis. The removal criterion was *F*-to-remove ≥ 0.10.

All statistical analyses were conducted using IBM® SPSS® Statistics 22 for Windows (IBM Corp., Armonk, NY, USA). Two-sided statistical significance was assumed at *p* values less than 0.05 with a 95% confidence interval.

## Results

**Fig. 1:**
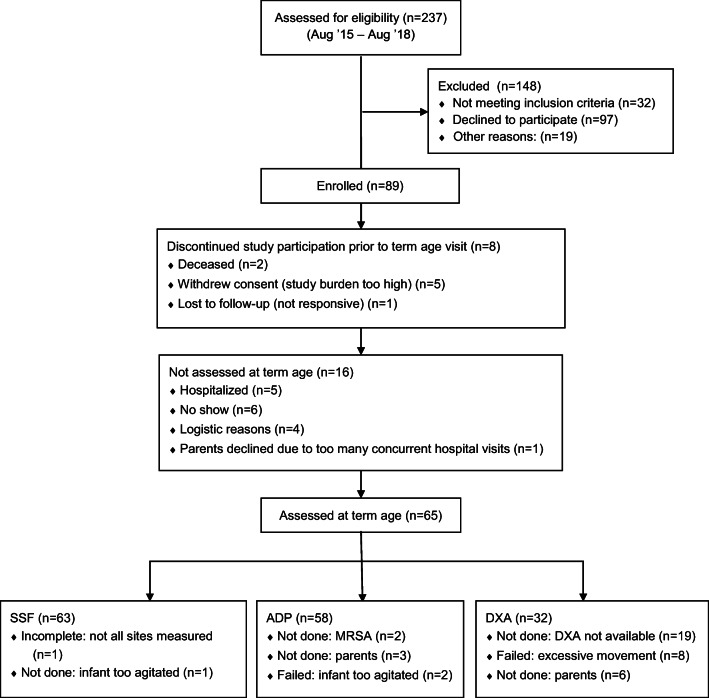
NUTRIE study flow diagram

Sixty-five infants were assessed for growth and body composition at term age. Baseline characteristics are shown in Table 1. Measurements of skinfolds were successfully completed in 63 infants; ADP was successful in 58 infants and DXA in 32 infants (see Fig. 1).

**Table 1:** Baseline characteristics

	Total, *n* = 65	Male, *n* = 35	Female, *n* = 30	*p* value^a^
Characteristics at birth				
Gestational age (weeks), mean ± SD	29.0 ± 1.6	29.3 ± 1.6	28.7 ± 1.6	0.132
Race, *n* (%)				
White	52 (80.0)	27 (77.1)	25 (83.3)	0.534
Non-white	13 (20.0)	8 (22.9)	5 (16.7)	
Birth weight (g), mean ± SD	1170 ± 316	1347 ± 295	1108 ± 293	0.002
Birth length (cm), mean ± SD	37.0 ± 3.2	38.6 ± 3.0	36.3 ± 3.0	0.004
Birth head circ. (cm), mean ± SD	26.3 ± 2.2	27.4 ± 2.3	26.1 ± 1.9	0.016
Birth weight SDS, median (IQR)	0.2 (− 0.3 to 0.6)	0.3 (− 0.1 to 0.9)	− 0.1 (− 0.4 to 0.5)	0.065
Birth length SDS, median (IQR)	0.3 (− 0.5 to 0.6)	0.3 (− 0.3 to 0.6)	0.1 (− 0.6 to 0.5)	0.229
Birth head circ. SDS, median (IQR)	0.4 (− 0.2 to 1.0)	0.3 (− 0.2 to 1.2)	0.4 (− 0.3 to 1.0)	0.246
Small for gestational age (< p10), *n* (%)	3 (4.6)	1 (2.9)	2 (6.7)	0.782
Characteristics at term age visit				
PMA at term age visit (weeks), mean ± SD	43.8 ± 1.9	43.8 ± 2.1	43.8 ± 1.6	0.962
Weight at term age visit (g), mean ± SD	4078 ± 662	4320 ± 683	3795 ± 517	0.001
Length at term age visit (cm), mean ± SD	52.8 ± 2.7	53.9 ± 2.4	51.5 ± 2.5	0.000
Head circ. at term age visit (cm), mean ± SD	37.4 ± 1.6	37.8 ± 1.6	37.0 ± 1.4	0.040
Weight SDS at term age visit, median (IQR)	− 0.5 (− 1.3 to 0.1)	− 0.2 (− 1.1 to 0.3)	− 0.7 (− 1.5 to 0.0)	0.104
Length SDS at term age visit, median (IQR)	− 0.4 (− 1.1 to 0.1)	− 0.2 (− 0.7 to 0.3)	− 1.0 (− 1.7 to − 0.2)	0.003
Head circ. SDS at term age visit, median (IQR)	0.5 (− 0.1 to 1.3)	0.7 (0.0 to 1.6)	0.3 (− 0.1 to 1.2)	0.272
Postnatal growth restriction^b^, *n* (%)	18 (27.7)	7 (20.0)	11 (36.7)	0.134
Type of nutrition				
Human milk	29 (44.6)	18 (51.4)	11 (36.7)	
Formula	36 (55.4)	17 (48.6)	19 (63.3)	

^a^*p* value for females compared with males based on t test for normal distributions, Mann-Whitney *U* test for non-parametric variables, and Pearson chi-square or Fisher’s exact test for categorical variables

^b^Postnatal growth restriction was assumed if, at 36 weeks postmenstrual age, there was a decrease in weight z-score of more than 1 SD compared with the birth weight z-score

*IQR* interquartile range, *PMA* postmenstrual age, *SD* standard deviation, *SDS* standard deviation score

### DXA compared with ADP

**Fig. 2:**
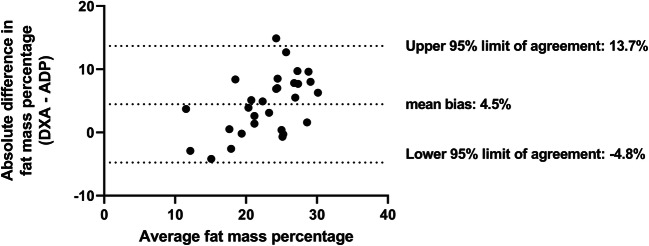
Fat mass percentage obtained via DXA compared with fat mass percentage obtained via ADP. ADP air displacement plethysmography, DXA dual-energy X-ray. Bland-Altman plot of fat mass percentage measured by DXA compared with ADP. Average of fat mass measured with DXA and ADP is depicted on the x-axis and the difference between the fat mass percentage measured with DXA and ADP is depicted on the y-axis. Mean difference: 4.5 ± 4.7%, lower limit of agreement: − 4.8%, upper limit of agreement: 13.7%, maximum allowed difference: 2%

Compared with ADP, fat mass measured with DXA was higher (254.7 g, 165.7–343.9). Likewise, fat mass percentage measured with DXA was 4.5% (2.7–6.2) higher than ADP (Table 2). There was no agreement between fat mass measured with DXA compared with ADP. The mean difference was 255 ± 234 g with a lower limit of agreement of − 212 g and an upper limit of agreement of 723 g. For fat mass percentage, the mean difference between DXA and ADP was 4.5 ± 4.7%, with a lower limit of − 4.8% and an upper limit of 13.7%. The Bland-Altman plot showed a proportional bias: as the mean fat mass percentage increased, the absolute difference in fat mass percentage between the two methods increased. Based on a maximum allowed difference of 2%, no agreement was found (Fig. 2). For both ADP and DXA, fat mass and fat mass percentage at term age did not differ significantly between gender, ethnicity, or type of nutrition at term age.

**Table 2:** Body composition at term equivalent age

	Total, *N* = 65	Male, *N* = 35	Female, *N* = 30	*p* value^a^
Sum of skinfolds (mm)^b^, mean ± SD	22.4 ± 4.2	22.4 ± 4.4	22.5 ± 4.1	0.872
Waist circumference (mm)^c^, median (IQR)	36.0 (34.0–37.5)	36.5 (34.5–37.8)	35.4 (33.3–37.0)	0.167
ADP fat mass (g)^d^, mean ± SD	864 ± 253	910 ± 280	799 ± 198	0.100
DXA fat mass (g)^e^, mean ± SD	1078 ± 417	1187 ± 475	1012 ± 376	0.258
ADP fat mass percentage, median (IQR)	20.7 (18.4–23.0)	21.2 (18.4–23.4)	20.4 (18.1–22.6)	0.594
DXA fat mass percentage, median (IQR)	25.0 (21.5–30.5)	25.1 (22.4–31.8)	24.8 (19.8–29.5)	0.684
ADP fat-free mass (g), mean ± SD	3309 ± 462	3447 ± 430	3114 ± 442	0.006
DXA fat-free mass (g), mean ± SD	3138 ± 400	3316 ± 422	3032 ± 356	0.051
ADP fat-free mass percentage, median (IQR)	79.3 (77.1–81.6)	78.8 (76.6–81.7)	79.7 (70.5–81.9)	0.594
DXA fat-free mass percentage, median (IQR)	75.1 (69.6–78.5)	74.9 (68.2–77.7)	75.2 (70.5–80.2)	0.686

^a^*p* value for females compared with males based on t test for normal distributions, Mann-Whitney *U* test for non-parametric variables, and Pearson chi-square or Fisher’s exact test for categorical variables

^b^Sum of skinfolds measured at biceps, triceps, subscapular, and supra-iliac positions, *n* = 63 (34 males, 39 females)

^c^Waist circumference, *n* = 63 (34 males, 39 females)

^d^ADP, *n* = 58 (34 males, 24 females)

^e^DXA, *n* = 32 (12 males, 24 females)

*ADP* air displacement plethysmography, *DXA* dual-energy X-ray, *IQR* interquartile range, *SD* standard deviation

### DXA compared with skinfolds

**Fig. 3:**
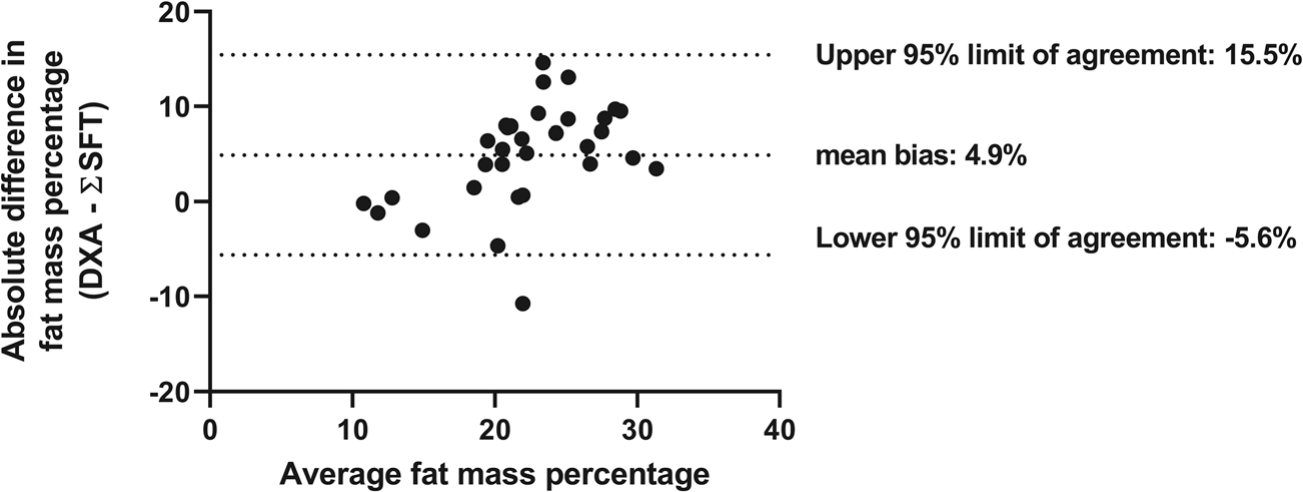
Fat mass percentage obtained via DXA compared with the estimated fat mass percentage based on ΣSFT. Bland-Altman plot of fat mass percentage measured by DXA compared with fat mass percentage estimated based on the formula by Schmelzle and Fusch [18]. Average of fat mass percentage measured with DXA and estimated with the formula is depicted on the x-axis and the difference between the fat mass percentage measured with DXA and estimated by the formula is depicted on the y-axis. Mean difference: 4.9 ± 5.4%, lower limit of agreement − 5.6%, upper limit of agreement 15.5%, maximum allowed difference 2%

The difference between fat mass estimated through the model of Schmelzle and Fusch and the fat mass measured with DXA exceeded the limits of agreement. The mean difference was 272 ± 240 g, with a lower limit of agreement of − 742 g and an upper limit of agreement of 199 g. Fat mass percentage derived from skinfolds did not agree with fat mass percentage measured with DXA and showed a proportional bias with a larger difference in fat mass percentage with increasing mean fat mass percentage (Fig. 3).

### Predictive model for fat mass measured with DXA

Within our cohort, fat mass, measured with DXA, could be estimated with gestational age, waist circumference, length, and the ∑SFT: fat mass (g) = − 4649.1 + 23.5*∑SFT + 64.4*length + 77.6*waist circumference − 33.7*gestational age (∑SFT in mm, length and waist circumference in cm, and gestational age in weeks). These factors explained 89% of the variance (*R*^2^ = 0.893, S.E. of the estimate 146 g, *p* < 0.001). In addition, 75% of the variance in fat mass percentage, measured with DXA, could be explained by waist circumference, head circumference, and the ∑SFT (*R*^2^ = 0.753, S.E. of the estimate 3.5%, *p* < 0.001).

### Predictive model for fat mass measured with ADP

Within our cohort, fat mass, measured with ADP, could be estimated with gestational age, waist circumference, head circumference, weight SDS, head circumference SDS, and the ∑SFT: fat mass (g) = − 3013.0 − 9.4*gestational age + 39.1*waist circumference + 65.9*head circumference + 67.6*weight SDS − 59.3*head circumference SDS + 15.1*∑SFT (gestational age in weeks, waist and head circumference in mm, and ∑SFT in mm). These factors explained 72% of the variance (*R*^2^ = 0.716, S.E. of the estimate 138.4 g, *p* < 0.001).

Forty-two percent of the fat mass percentage measured with ADP could be explained by the ΣSFT and waist circumference (*R*^2^ = 0.426, S.E. of the estimate 3.1%, *p* < 0.001)

In multivariate analysis, other potential confounders were found to not be significant.

## Discussion

This study showed that there is poor agreement between body composition measured with ADP and body composition measured with DXA in preterm born infants at term equivalent age. Compared with ADP, DXA showed higher fat mass percentages. Furthermore, estimations of fat mass based on the ∑SFT showed poor agreement with the actual fat mass measured with DXA.

Various studies in term infants report high correlations between fat mass measured with ADP and fat mass measured with DXA [9, 10]. Nevertheless, a high correlation does not imply both methods found the same value and does not provide information about the test quality [24]. Similar to studies performed in full-term infants, DXA gave higher estimates of fat mass in our cohort compared with ADP [10, 9]. In agreement with that, early animal studies showed that DXA seems to overestimate fat mass [25, 26]. To our knowledge, no data has been published on the comparison of DXA and ADP in extremely preterm infants. Nevertheless, one recent study in South-African term infants also showed higher estimates of fat mass by ADP compared with DXA [8]. Moreover, several reviews have highlighted that both DXA and ADP have reasonable reproducibility, but only modest accuracy. According to these reviews, ADP actually seems to underestimate fat-free mass percentage or fat-free mass expressed in grams per liter (fat-free mass density). Especially, when the fat-free mass percentage or density gets higher, the underestimation becomes larger [27, 15]. In actual fact, the fat-free mass percentage or density may be a more relevant parameter to assess, as in practice it may be more insightful to properly predict fat mass and fat-free mass percentage than it is to predict absolute fat and fat-free mass. Nonetheless, in all these studies, it is to be questioned whether an appropriate reference method for body composition has been used. In practice, both DXA and ADP, as well as deuterated water, have been deemed as reliable methods; however, there seems to be no universally accepted preferential reference method in living infants.

In contrast to DXA, ADP takes into account that hydration status is different in infants as compared with that in adults. Particularly during the first week of life, infants’ fat-free mass hydration is higher, and therefore, DXA estimations of fat and fat-free mass may not be as accurate as ADP estimations in this period [28]. Moreover, the algorithms used in DXA software are not open for critical analysis. In addition, DXA quality is negatively influenced by movement, while moderate movement does not affect body composition measurements taken using ADP. Moreover, infants are exposed to a low dose of radiation. Therefore, in this study, and presumably in others as well, ADP measurements may have been more reliable.

In line with others, we could not externally validate the model by Schmelzle et al. for fat mass prediction [29]. To date, predictive models for the estimation of fat mass using the SFT have only been validated in term and late preterm infants and the predictive value of SFT alone was generally low [16, 30]. Moreover, these models were mostly only internally validated and looked at the prediction of absolute fat mass and not fat mass percentage [13, 12, 29]. However, in our cohort, it seems that the ∑SFT could also explain an important part of the variance in fat mass percentage. Even though the prediction model yielded a lower *R*^2^ than the model for the prediction of fat mass, fat mass percentage may be a more generalizable factor and worth further exploration for external validation. Especially, in the light of resource poor settings, the ∑SFT might still be useful as an indicator of fat mass percentage.

Our study was limited by the small sample size, which reduces the generalizability of the prediction models. Moreover, body composition was measured between 38 and 46 weeks postmenstrual age, a period in which body composition alters [31]. In addition, the low number of successful DXA scans, with 1 in 4 scans not completed because of excess movement or too much agitation prior to the measurement, limited the assessment of agreement between different methods. Recent studies in term infants have shown that placing infants in a vacuum cushion limits movement artifacts and leads to more comparable results between DXA and ADP [32].

This study has not been able to robustly show that skinfold measurements qualify as a reliable, low-cost point-of-care instrument. However, it remains desirable to find an easily accessible and reliable way of monitoring fat mass in light of possible adverse cardiometabolic outcomes in later life [1–3]. Nevertheless, currently available methods for bedside assessment of body composition, such as bioelectrical impedance analysis and body proportionality measures, have a questionable accuracy and accurate, low-cost bedside methods are limited [30]. To the best of our knowledge, other predictive equations including weight and length indices and easily measured clinical parameters are yet to be externally validated [33–35]. It would be of interest to further investigate the potential of these predictive equations. Taking previous findings into account, ADP seems to be more practical to assess body composition, in particular fat mass, in preterm infants in early life. Nevertheless, it remains to be elucidated whether or not a DXA without movement artifacts provides a more accurate assessment of whole body composition than ADP in preterm infants.

## Abbreviations

ADP: Air displacement plethysmography
DXA: Dual-energy X-ray
PMA: Postmenstrual age
SDS: Standard deviation score
SGA: Small for gestational age
